# Evolution of tetraspanin antigens in the zoonotic Asian blood fluke *Schistosoma japonicum*

**DOI:** 10.1186/s13071-023-05706-3

**Published:** 2023-03-14

**Authors:** Daniel A. J. Parsons, Anthony J. Walker, Aidan M. Emery, Joanne P. Webster, Scott P. Lawton

**Affiliations:** 1 Molecular Parasitology Laboratory, School of Life Sciences, Pharmacy and Chemistry, Kingston University London, Kingston Upon Thames, Penrhyn Rd, Surrey, KT1 2EE UK; 2 Department of Life Sciences, Natural History Museum, Cromwell Rd, South Kensington, London, SW7 5BD UK; 3 Department of Pathobiology and Population Sciences, Royal Veterinary College, University of London, Hawkshead Campus, Herts, AL9 7TA UK; 4 Centre for Epidemiology & Planetary Health, Department of Veterinary and Animal Sciences, Northern Faculty, Scotland’s Rural College, An Lóchran, 10 Inverness Campus, Inverness, IV2 5NA UK

**Keywords:** *Schistosoma japonicum*, China, Tetraspanin, Vaccine candidate, Selection pressures, Immune evasion, Antigenic variability

## Abstract

**Background:**

Despite successful control efforts in China over the past 60 years, zoonotic schistosomiasis caused by *Schistosoma japonicum* remains a threat with transmission ongoing and the risk of localised resurgences prompting calls for a novel integrated control strategy, with an anti-schistosome vaccine as a core element. Anti-schistosome vaccine development and immunisation attempts in non-human mammalian host species, intended to interrupt transmission, and utilising various antigen targets, have yielded mixed success, with some studies highlighting variation in schistosome antigen coding genes (ACGs) as possible confounders of vaccine efficacy. Thus, robust selection of target ACGs, including assessment of their genetic diversity and antigenic variability, is paramount. Tetraspanins (TSPs), a family of tegument-surface antigens in schistosomes, interact directly with the host’s immune system and are promising vaccine candidates. Here, for the first time to our knowledge, diversity in *S. japonicum* TSPs (SjTSPs) and the impact of diversifying selection and sequence variation on immunogenicity in these protiens were evaluated.

**Methods:**

SjTSP sequences, representing parasite populations from seven provinces across China, were gathered by baiting published short-read NGS data and were analysed using in silico methods to measure sequence variation and selection pressures and predict the impact of selection on variation in antigen protein structure, function and antigenic propensity.

**Results:**

Here, 27 SjTSPs were identified across three subfamilies, highlighting the diversity of TSPs in *S. japonicum*. Considerable variation was demonstrated for several SjTSPs between geographical regions/provinces, revealing that episodic, diversifying positive selection pressures promote amino acid variation/variability in the large extracellular loop (LEL) domain of certain SjTSPs. Accumulating polymorphisms in the LEL domain of SjTSP-2, -8 and -23 led to altered structural, functional and antibody binding characteristics, which are predicted to impact antibody recognition and possibly blunt the host’s ability to respond to infection. Such changes, therefore, appear to represent a mechanism utilised by *S. japonicum* to evade the host’s immune system.

**Conclusion:**

Whilst the genetic and antigenic geographic variability observed amongst certain SjTSPs could present challenges to vaccine development, here we demonstrate conservation amongst SjTSP-1, -13 and -14, revealing their likely improved utility as efficacious vaccine candidates. Importantly, our data highlight that robust evaluation of vaccine target variability in natural parasite populations should be a prerequisite for anti-schistosome vaccine development.

**Graphical abstract:**

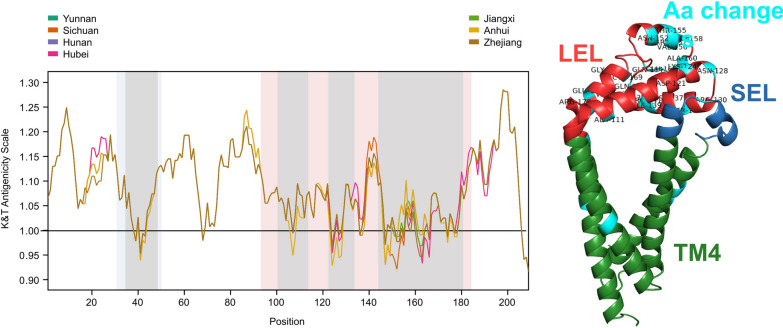

**Supplementary Information:**

The online version contains supplementary material available at 10.1186/s13071-023-05706-3.

## Background

Across Southeast Asia, zoonotic schistosomiasis, caused by *Schistosoma japonicum*, is responsible for infection of humans, as well as ~ 40 species of wild and domesticated animals, where animal hosts play an important role in maintaining parasite transmission [1, 2]. The recent Healthy China 2030 strategic plan outlines a revised schistosomiasis elimination target of 2030, in line with the recent WHO NTD roadmap [3, 4]. However, despite well-concerted, multi-disciplinary control efforts over the last 60 years [5, 6], which have resulted in a reduction in human infections from > 11 million people to ~ 30,000, as well as a similar reduction in livestock infections [7, 8], the risk of resurgence remains ever present in China and other schistosome-endemic SE Asian countries and is expected to increase, in part due to persistent hotspots of infection, the perpetual presence of refugia, and anthropogenic changes to the climate and environment [9–12].

The development of an effective anti-schistosome vaccine, which has recently seen renewed interest globally, would provide an essential tool in moving towards end-game elimination in China. Concerns of reduced praziquantel (PZQ) susceptibility would be mitigated by implementing a vaccine in current control strategies, complementing and reducing reliance on frequent PZQ mass drug administration (MDA) and thereby simultaneously supressing the risk of parasites evolving PZQ resistance [13–15], thus serving to reduce the possibility of future resurgences [10, 16–18].

To date, more than 100 vaccine candidate antigens have been identified and tested against various schistosome species within animal models [17]. A protein family of particular interest, the transmembrane, tegument- and oesophageal-associated tetraspanins (TSPs), have been shown to elicit variable levels of immune response in animal models, including murine and bovine definitive hosts [19–22].

Of the known *S. japonicum* TSPs (SjTSPs), several have been shown to interact directly with host immune components via their large extracellular loop (LEL) domains [23–26], with a number of TSPs shown to be crucial in tegument development, maturation and stability [27], and which are also involved in host immune evasion, transition to parasitism and survival in the definitive host [23, 24, 28–31]. Several TSPs have been suggested as anti-schistosome vaccine candidates, particularly SjTSP-2 and -23 [17, 23, 25, 29–32]. However, despite the immunological properties of SjTSPs supporting their use as vaccine targets, only partial protection and variable efficacy have been demonstrated in animal-based SjTSP immunisation studies [19, 20, 30].

The TSP-LEL domain is highly polymorphic, particularly in the case of SjTSP-2, with such ‘hypervariability’ suggested to underly the variable vaccine efficacy [24, 26, 30, 33, 34]. Indeed, the polymorphic nature and genetic diversity of TSP-2 and TSP-23, demonstrated within and between schistosome species, have been suggested to limit their utility as vaccine targets [19, 24, 25, 30, 32, 35]. Schistosomes express multiple TSP variants through alternative splicing and allelic variation [24, 30, 34, 36], and hypervariability within the TSP-LEL domain is suggested to reflect the selective influence of the definitive host, resulting in antigenic variation [33].

Elucidating how antigen diversity and evolution influence the antigenic propensity of SjTSP proteins remains necessary to properly evaluate their utility as vaccine targets [32–34]; however, these aspects of SjTSP biology have not been explored. Here, by employing in silico approaches using TSPs retrieved from published *S. japonicum* genomic datasets, this study aimed to identify putative SjTSPs and determine their diversity and evolutionary history in *S. japonicum*. In particular, the geographic variability of SjTSP sequence from seven provinces across China was assessed, with the aim of identifying which members of the antigen family were most variable, localising particularly variable SjTSP regions and categorisng selection pressures driving the measured SjTSP geographical sequence variation. The results obtained should have significant theoretical and applied implications, particularly regarding the identification of future anti-schistosome vaccine targets.

## Methods

### SjTSP sequence retrieval, characterisation and phylogenetic analysis

Owing to the paucity of available and accurately described tetraspanin sequence data from *S. japonicum*, the full SjTSP protein family remains poorly resolved. Thirty-three putative SjTSP mRNA sequences were retrieved from the *S. japonicum* genome assembly on WormBase ParaSite [37], and a further 29 were identified from literature sources (Table 1) [29, 32, 38]. Where coding sequences (cds) were absent from the literature and online databases, whole gene and protein sequences were queried in GeneWise [39] to retrieve the related cds.

**Table 1:** Tetraspanin sequence sources and related domain information

Tetraspanin	Accession number	Amino acid residue positions			Source
		Total	SEL	LEL	
TSP-1	CAX70118.1	247	36–49	107–202	[85]
TSP-2	AEG74374.1	215	35–53	106–183	[30]
TSP-8	AAW24708.1	224	36–54	107–189	[86]
TSP-13	CAX69650.1	233	35–43	93–194	[85]
TSP-14	CAX69587.1	270	32–72	125–235	[85]
TSP-23	CAX72085.1	218	36–49	108–183	[85]
TSP-25	AY814252.1	287	101–115	166–252	[86]

*SEL* small extracellular loop, *LEL* large extracellular loop

Putative TSP protein sequences were validated using TMHMM 2.0 to confirm their membrane association, SMART to predict domain architecture (presence of the two extracellular loop domains [26] and PROTTER to assess protein membrane topology [40–42]. From these structurally validated SjTSPs, seven were selected for detailed analysis (Table 1) based upon an analysis of published information pertaining to each SjTSPs structure, function, immunogenicity and vaccine candidacy [20, 25, 26, 32, 38].

Structurally validated SjTSP protein sequences were aligned using the MEGA7-integrated MUSCLE tool [43, 44], and phylogenetic reconstruction was done using the distance-based neighbour joining (NJ) method [45] to assess the relationships between members of the *S. japonicum* tetraspanin family. Nodal support was determined using 1000 bootstrap replicates. Amino acid positions containing gaps and missing data were omitted from analysis.

### Genetic variation of SjTSPs and identification of selection pressures

The seven SjTSP reference sequences (Table 1) were used to bait short-read NGS data, sequenced from ten pairs of adult worms from each of the seven sampled provinces across China (Yunnan, Sichuan, Hunan, Hubei, Jiangxi, Anhui and Zhejiang) (Additional File 1: Fig. S1) by Young et al. [34]. Short-read NGS data were reported by Young et al. [34] to have achieved up to 50-fold coverage of the *S. japonicum* reference genome for each of the seven sampled parasite populations, considered to be sufficient for draft genome assembly, and the identification of putative protein coding genes for population and phylogenetic analysis. Using a similar approach as seen in [46, 47], sequence read archive (SRA)-BLAST was employed to retrieve the complete cds for each provincial SjTSP variant [48]. SjTSP SRA-collected sequences were aligned against SjTSP reference sequence and assembled using the Contig Assembly Program 3 (CAP3) feature in Bioedit [49, 50]. Finally, using MUSCLE, multiple sequence alignments were constructed representing parasite isolates from each of the Chinese provinces for each SjTSP.

Phylogeographic relationships, also analysed using MEGA7, were investigated using provincial SjTSP cds alignments, inferred using the NJ method and 500 bootstrap replicates. *Schistosoma mansoni* and *Schistosoma haematobium* TSP orthologues were used to root each phylogeny. Orthologous Sm- and ShTSPs were gathered through a BLASTn similarity search [51] of each SjTSP against the NCBI nucleotide collection, filtering for hits against each *Schistosoma* species.

Measures of genetic diversity were calculated using DNASP v6.12 [52] to gain insight into the geographic variation of SjTSPs between distinct *S. japonicum* populations across China, such as the number of polymorphic sites, number of haplotypes (h), haplotype diversity (Hd) and nucleotide diversity (π).

To detect the presence and extent of selection acting on the SjTSPs, non-synonymous (dN) and synonymous (dS) substitutions, averaged across all codons in the alignment (ω) for the whole cds and LEL domain encoding regions, were determined using MEGA7. Conventional thresholds for inferring the occurrence and extent of selection were used (positive selection, ω > 1; purifying selection, ω < 1; neutrality, ω = 1). The effect of selection on protein-coding genes is often difficult to identify, as traditional models, such as ω, predict selection across all sites in an alignment and thus lack statistical power, as selection may only occur at a small number of sites [53, 54]. To define individual nucleotide sites under selection, and thus increase the resolution of the ω analysis, the Mixed Effects Model of Evolution (MEME) [55] and Fast, Unconstrained Bayesian AppRoximation (FUBAR) [54] analyses were performed via Datamonkey [56]. MEME applies a mixed-effects maximum-likelihood approach to test for episodic and diversifying selection; *P*-value thresholds were set to 0.5. FUBAR employs a Bayesian approach to infer positive or purifying selection at each site across the SjTSP alignment. As default, posterior probability value > 0.9 strongly indicates the presence of positive or purifying selection at a site.

### Determining the effects of sequence variation on protein structure and function

Using BioEdit, SjTSP sequences from each of the seven provinces were translated into proteins to determine the impact of accumulating polymorphisms on each SjTSP amino acid sequence. Amino acid changes and B-cell antibody binding sites (epitopes), predicted using BepiPred-2.0 [57], were mapped onto SjTSP protein secondary structures and visualised using PROTTER.

To generate 3D protein models of each SjTSP, Protein Data Bank (PDB) models were generated from reference sequences using I-TASSER [58]. Confidence scores (c scores) estimated the quality of the models (> −5 = high confidence). Subsequently, PyMOL [59] was used to render 3D structures of the I-TASSER-produced SjTSP reference PDB files, enabling comprehensive visualisation of key domains, antibody binding sites and sequence variation in relation to protein tertiary structure.

Structural and conformational variation between provincial SjTSPs was assessed by comparing protein structures within DALI [60]. DALI *z* scores that define structural homology between provincial SjTSP 3D protein models were then used to evaluate the evolutionary divergence and clustering between the proteins using principal component analyses (PCAs).

To determine whether amino acid substitutions impacted the function of each SjTSP protein, SjTSP reference sequences and site location information of amino acid substitutions were uploaded into PROVEAN [61], generating a PROVEAN score. A score < −2.5 was used to predict whether an amino acid substitution alters SjTSP function at that individual site. TreeSAAP v3.2 was then used to further investigate selection and determine whether amino acid changes significantly altered the physicochemical properties of each SjTSP [62], producing *z* scores to establish the significance of amino acid replacement, type of deviation from neutrality and ranking the magnitude of changes in 31 physiochemical amino acid properties [63, 64]. Eight magnitude categories were analysed, with higher categories (5–8) denoting the most radical changes in protein biochemistry. Significantly positive or negative *z* scores (> ± 1.645 = *P* < 0.05) indicate the influence of positive or purifying selection, respectively [64]. Significant and radical changes were considered evidence of positive selection resulting in adaptation (magnitude categories 5–8, *z* scores > 3.09 = *P* < 0.001).

### Evaluating the impact of selection and provincial SjTSP variation on antigenicity and antibody binding affinity

SjTSP protein sequences were compared between provinces to determine the association between sequence diversity and antigenicity, using the Kolaskar and Tongaonkar (KT) method in the IEDB Antibody Epitope prediction tool [65]. The KT method is one of the most accurate, robust and widely used predictors of antigenicity of proteins. It applies an accurate semi-empirical approach by utilising the physiochemical properties of amino acid residues and relative frequency of experimentally determined epitopes to predict antigenic determinants at each site directly from a protein sequence [66].

To elucidate effects of amino acid substitutions on antibody binding affinity in each SjTSP, mCSM-Ab was employed [67]. Utilising SjTSP 3D protein models and amino acid substitutions, mCSM-Ab employs a graph-based machine-learning approach to infer changes in structural signatures from each residue in the SjTSP model that resulted from amino acid variation. Predictions of the difference in Gibbs free energy (ΔΔ*G* in Kcal/mol) between reference SjTSP 3D protein models and provincial SjTSPs with amino acid substitutions were established to accurately assess the effects of mutations on the propensity for antibody binding.

## Results

### Phylogenetic relationship of TSP proteins in *Schistosoma japonicum*

**Fig. 1:**
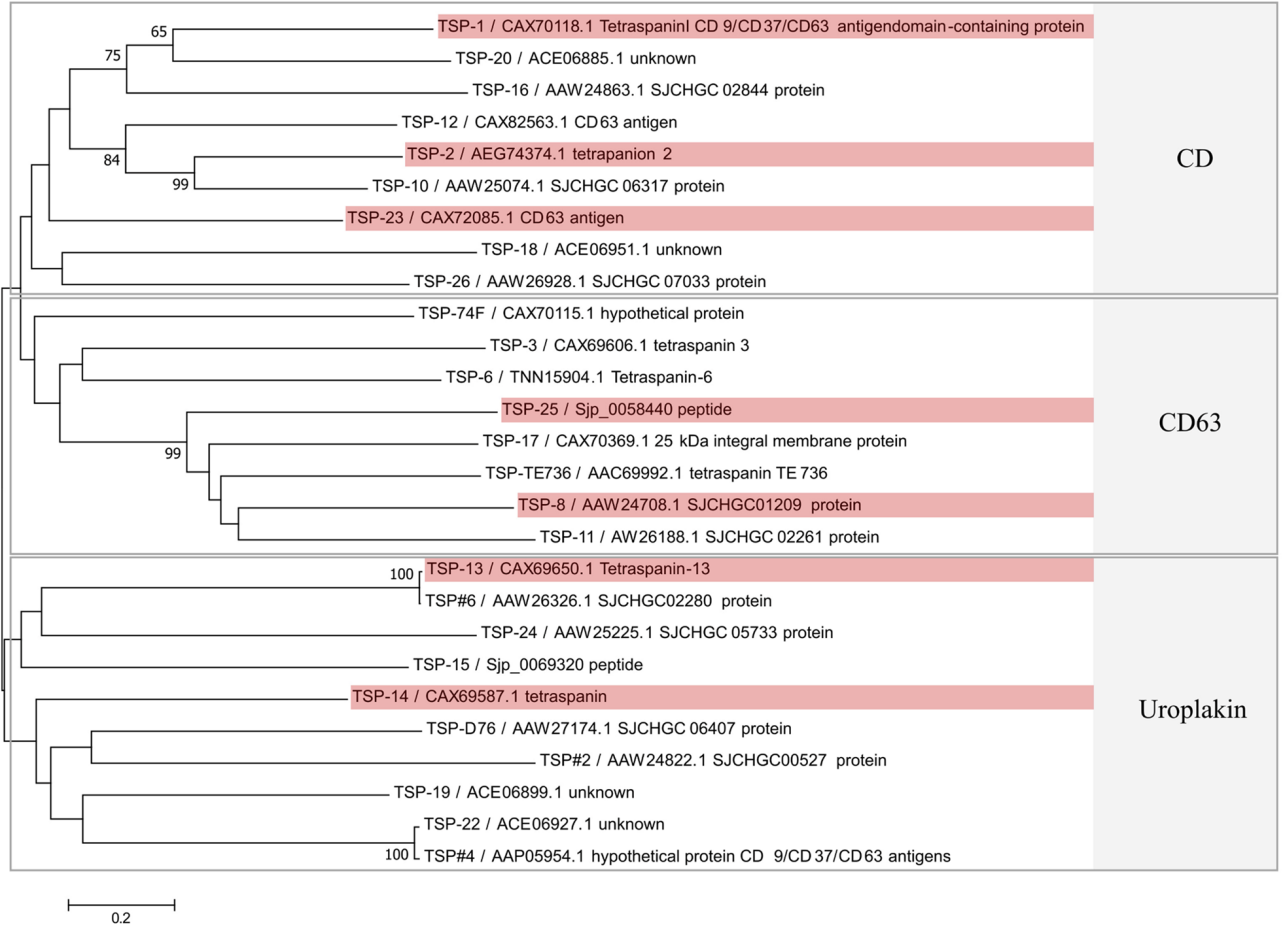
Phylogenetic analysis of 27 protein sequences from the tetraspanin (TSP) protein family in *Schistosoma japonicum*. Evolutionary history was inferred using the neighbour-joining method, with 1000 bootstrap replicates, using MEGA7. Bootstrap values with > 50% support are displayed at each node. Three major SjTSP subfamily clades are highlighted in grey; highlighted sequences (red) were utilised for further analyses

From 62 sequences annotated as SjTSPs, 27 were structurally validated as TSPs and were thus included for phylogenetic analysis. Phylogenetic reconstruction revealed that the TSP family in *S. japonicum* is highly divergent, with SjTSPs forming three monophyletic clades (Fig. 1). The clades resolved here correspond to the CD, CD63 and Uroplakin groupings, as described in other organisms, and in *S. japonicum* [29]. Deeper ancestral nodes, however, had low bootstrap support, providing uncertainty in the deeper tree topology. Only seven nodes demonstrated bootstrap support values > 50 after 1000 iterations. Clustering of SjTSP proteins in the CD subfamily was best supported, with four of the seven well-supported nodes evident in this clade. The short branch lengths and strong bootstrap support demonstrated in Fig. 1 for SjTSP-13 and TSP-6, and SjTSP-22 and TSP-4, suggest these SjTSPs are the same proteins.

### Genetic differentiation and diversity of tetraspanin proteins between *Schistosoma japonicum* populations

The seven SjTSPs selected for phylogeographic analysis and assessment of genetic and antigen geographic variation were primarily chosen based on their recognition as potentially protective antigens and therefore as promising vaccine targets.

**Fig. 2:**
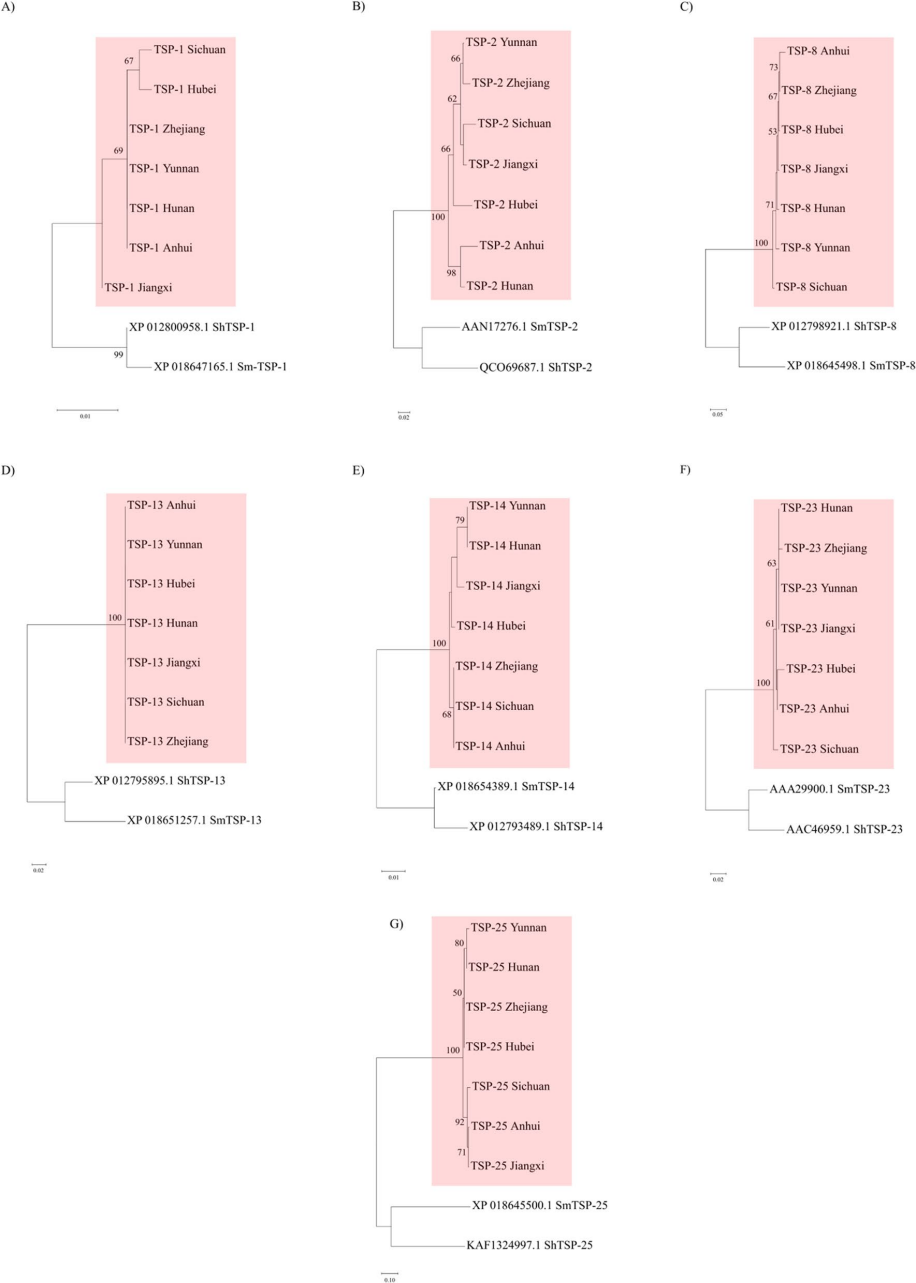
Phylogeographic analyses of provincial *Schistosoma japonicum* protein isolates (from Yunnan, Sichuan, Jiangxi, Zhejiang, Hunan, Hubei and Anhui) from seven TSP proteins: **A** SjTSP-2, **B** SjTSP-8, **C** SjTSP-23, **D** SjTSP-1, **E** SjTSP-13, **F** SjTSP-14, **G** SjTSP-25. Phylogenetic relationships were determined using the neighbour-joining method, with 500 bootstrap replicates, using MEGA7. Only bootstrap values with > 50% support are displayed at each node

Within all phylogeographic analyses, deeper ancestral nodes were generally well supported by bootstrap values (Fig. 2), supporting the resolved branching. Reconstructed phylogeographic relationships mostly divide SjTSP sequences into two distinct clades, other than for SjTSP-13; however, there was no consistent pattern based upon geographical distance between provinces (Fig. 2).

Nucleotide diversity (π) within *S. japonicum* populations was lowest for SjTSP-1, 13 and -14 (Table 2), and no allelic variants were found in the SRA data. In contrast, SjTSP-2, -25 and -8 were the most genetically diverse across the seven provinces. The varied π is also reflected in the haplotype diversity (Hd) for the seven STSPs, which ranged between 0.29 and 1. SjTSP-2 and -8 both displayed a Hd of 1, indicating each provincial sequence is a unique haplotype variant. SjTSP-13 demonstrated the lowest Hd compared to the other SjTSPs, with only two haplotypes shared across all seven analysed provinces (Table 2). Despite demonstrating one of the lowest π between provinces, SjTSP-1 had a Hd of 0.95, with six unique haplotypes that differed in only a few amino acids (Table 2). Between four (SjTSP-13) and 67 (SjTSP-2) polymorphic nucleotide sites were discovered across each SjTSP population. As such, SjTSP-13, -1 and -14 were shown to be relatively invariable between the seven provinces compared to the other SjTSPs (Table 2).

**Table 2:** Genetic diversity measurements for seven *Schistosoma japonicum* tetraspanin (TSP) metapopulations

Tetraspanin	Polymorphic sites	Nucleotide diversity (π)	π S.D	Number of haplotypes	Haplotype diversity (Hd)
SjTSP-1	5	0.0027	0.00056	6	0.95
SjTSP-2	67	0.0425	0.00936	7	1.0
SjTSP-8	19	0.0099	0.00212	7	1.0
SjTSP-13	4	0.0016	0.00112	2	0.29
SjTSP-14	5	0.0034	0.00057	4	0.81
SjTSP-23	8	0.0044	0.00096	5	0.86
SjTSP-25	27	0.0137	0.00226	6	0.95

### Signatures of selection in SjTSPs

**Fig. 3:**
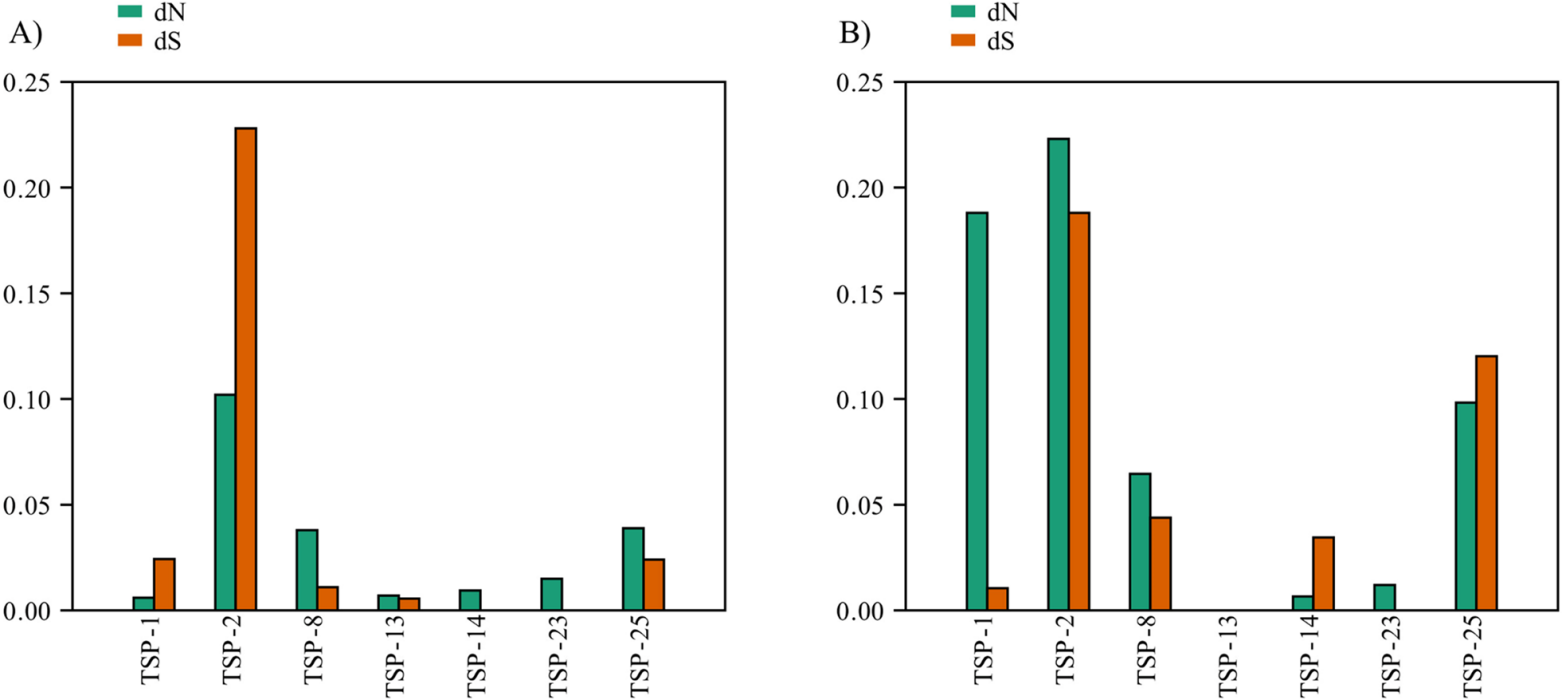
Signatures of selection acting upon seven tetraspanin (TSP) genes in *Schistosoma japonicum*. **A** The mean distribution of non-synonymous (dN) and synonymous (dS) mutations per TSP, averaged among all codons across the whole gene, or **B** among all codons within the region encoding the large extracellular loop (LEL) domain only. Mutations were estimated across all codons using DNASP and were plotted using PAST [82]

Substantial sequence variation was demonstrated within several SjTSPs from different provinces (Table 2); therefore, the impact of selection pressures was investigated for each SjTSP using ω. Mean dS and dN polymorphisms across the whole coding sequence (cds) were greatest for SjTSP-2, at 0.228 and 0.102, respectively (Fig. 3A), and were consistent with the far greater number of polymorphic sites detected in SjTSP-2 than the other SjTSPs (Table 2). SjTSP-25 has the second greatest dN and dS, both across the whole cds and LEL (Fig. 3), reflecting the number of detected polymorphic sites between provinces (Table 2). When assessing the whole cds, dS across SjTSP-1 and 2 is substantially higher than dN (Fig. 3A). In contrast, all other SjTSPs had higher dN than dS across each of their coding sequences. SjTSP-8, in particular, had > twofold higher dN compared to dS across the whole cds. When considering only the region encoding the LEL domain, SjTSP-2 also displayed the greatest dN and dS, where dN was more than twofold greater than dS (Fig. 3B), and relative to the remaining SjTSPs, dN and dS values were comparatively low. Overall, SjTSP-1, -2, -8 and -25 revealed an increase in dN, to varying degrees, when solely analysing the LEL cds (Fig. 3B), indicating the majority of nucleotide base changes that result in changes to the protein are accumulating in this important region of the gene.

Comparing the type and extent of selection for each SjTSP, using the ω averaged across all codons, analysing the LEL only or across the whole cds, considerable variation in ω was identified, both within and between SjTSPs (Table 3). The ω analysis indicates positive selection is acting on the LEL of SjTSP-2 and -8. Conversely, average ω values across the whole cds of SjTSP-2 revealed the presence of purifying selection, suggesting that positive selective pressures are disproportionately acting on the LEL domain. In contrast, SjTSP-25 showed signatures of positive selection across the whole cds, and despite increased dN and dS across the LEL, purifying selection was detected. Interestingly, SjTSP-8 displayed positive selection when considering both the whole cds and that of the LEL, although ω for the whole cds was ~ 2.3-fold greater than that of the LEL, suggesting that selection pressures have had a greater impact on regions outside the LEL domain in SjTSP-8.

**Table 3:** Signatures of selection, measured as mean dN/dS (ω) across all codons of the whole coding sequence and the region encoding the large extracellular loop (LEL) domain, within *Schistosoma japonicum* tetraspanins

Tetraspanin	Whole coding sequence	Region encoding large extracellular loop (LEL) domain
SjTSP-1	0.247	0
SjTSP-2	0.447	1.186
SjTSP-8	3.455	1.472
SjTSP-13	1.25	0
SjTSP-14	0.009	0.192
SjTSP-23	0.015	0.012
SjTSP-25	1.621	0.818

MEME and FUBAR were utilised to determine specific sites where selection may be acting (Additional File 2: Table S1). For SjTSP-2, MEME detected 21 sites under positive selection, 17 of which were within the LEL. FUBAR detected six sites under positive selection in SjTSP-2 (four in the LEL) and 13 under purifying selection (five with in the LEL). MEME and FUBAR both predicted positive selection at four sites (124, 128, 143 and 158), all of which reside within the LEL of SjTSP-2. Analysis of SjTSP-8 revealed seven sites under positive selection, three of which were within the LEL, whereas FUBAR predicted three sites under positive and one under purifying selection. Agreement was seen for three sites (84, 130 and 155), two of which are in the LEL. For SjTSP-23, six (MEME) and one site(s) (FUBAR) were considered under positive selection, with nucleotide site 169, contained within the LEL, called by both approaches. FUBAR failed to detect any purifying selection in SjTSP-23.

### In silico analysis of SjTSP protein structure

**Fig. 4:**
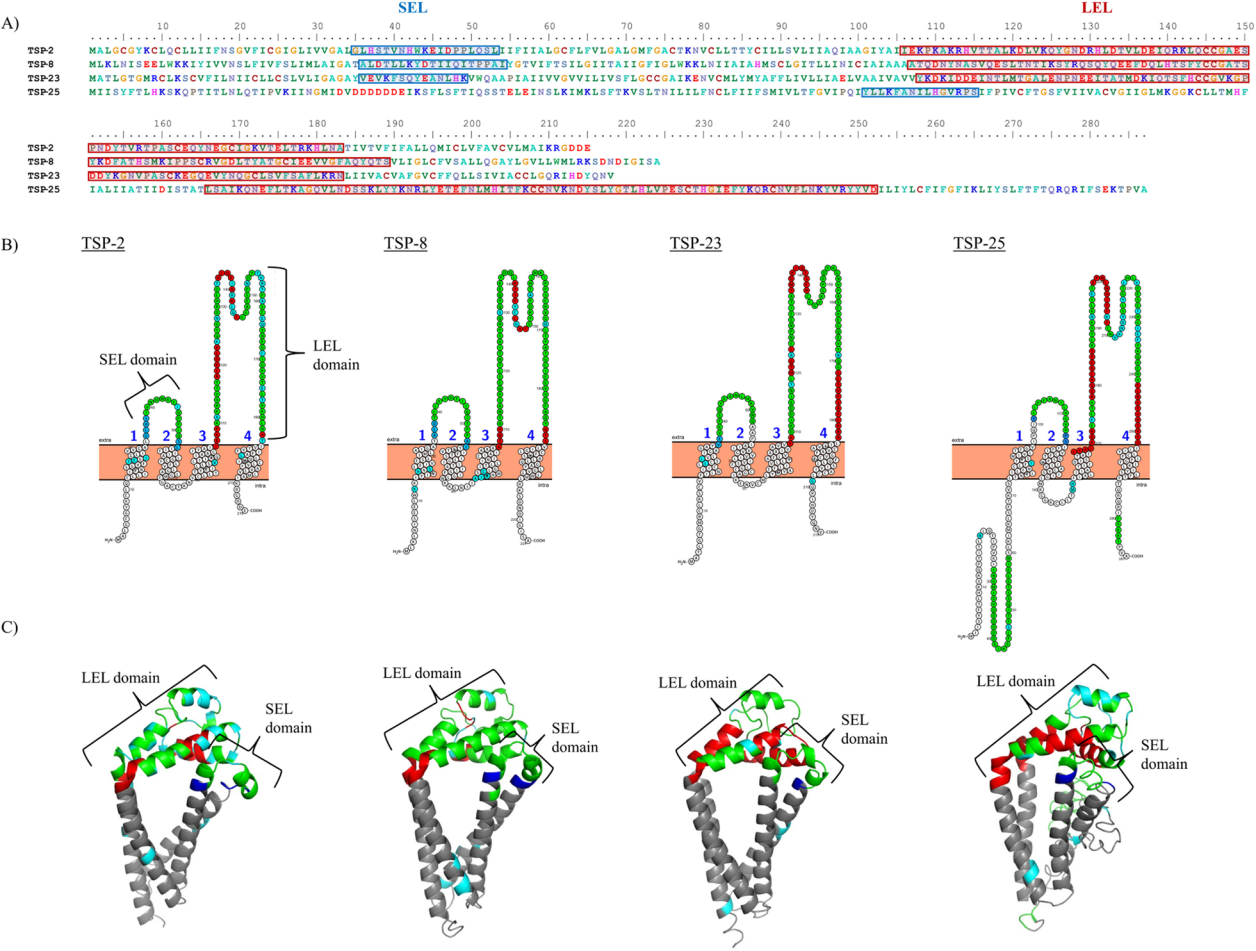
Primary (**A**), secondary (**B**) and tertiary structures (**C**) of *Schistosoma japonicum* tetraspanins (TSPs), SjTSP-2, SjTSP-8, SjTSP-23 and SjTSP-25. In **A**, **B** and **C** blue identifies the SEL domain and red the large extracellular loop (LEL) domain. In **B** and **C** green regions show the predicted epitopes and cyan the identified amino acid substitutions. Primary structure was visualised using BioEdit, secondary structure was predicted using PROTTER, and PyMOL molecular visualisation software was used to render the SjTSP tertiary structures

All analysed SjTSP proteins contained short cytoplasmic tails at the C- and N-termini, a small extracellular loop (SEL) domain, a LEL domain, four transmembrane helices and (with the exception of SjTSP-25) the conserved -CCG- (-Cys-Cys-Gly-) motif characteristic of the antigen family (Fig. 4). All seven 3D SjTSP protein models had c scores ≥ –2.13 (SjTSP-25), providing confidence in the I-TASSER-constructed SjTSP protein models. SjTSPs ranged from between 215 and 287 amino acids in length, with the SEL domains between 13 (SjTSP-1 and SjTSP-23) and 32 (SjTSP-14) and LEL domains between 77 (SjTSP-2) and 110 (SjTSP-14) amino acids (Table 1). According to BepiPred, several predicted B-cell epitopes were < 20 amino acids and thus of inadequate length to bind antibodies. All viable B-cell epitopes were predicted to exist in the extracellular loop domains (Fig. 4).

**Fig. 5:**
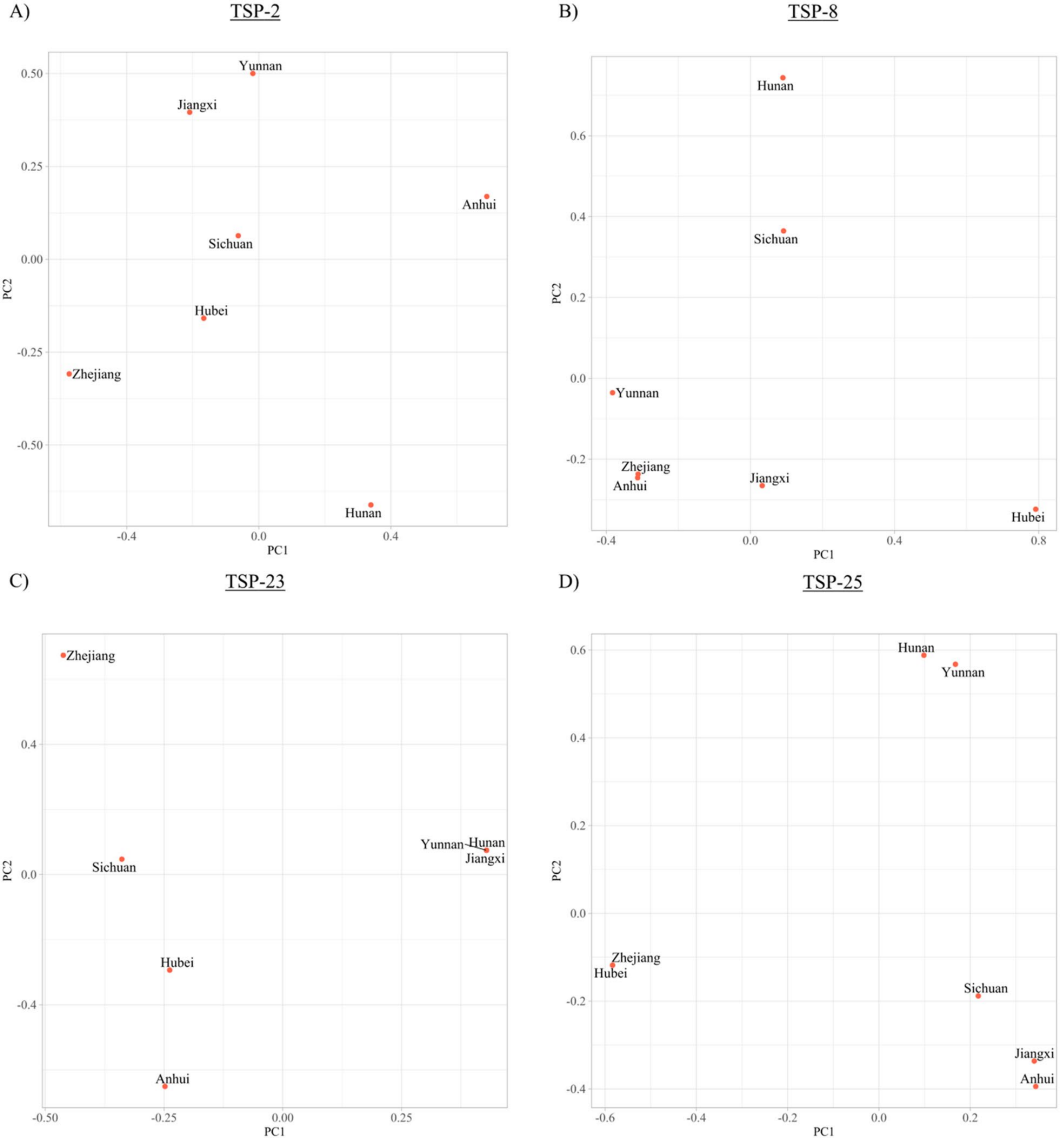
Principal component analyses of structural similarity for *Schistosoma japonicum* tetraspanin (TSP) proteins between provinces for SjTSP-2 (**A**), SjTSP-8 (**B**), SjTSP-23 (**C**) and SjTSP-25 (**D**). Structural similarity was inferred from *z* scores produced by the DALI protein comparison server and then plotted using the Tidyverse package [83] in RStudio [84]

Polymorphisms in the provincial SjTSPs translated to three amino acid substitutions in SjTSP-1, 34 in SjTSP-2, 17 in SjTSP-8, two in SjTSP-13, four in SjTSP-14, six in SjTSP-23 and 21 in SjTSP-25. Due to limited nucleotide diversity (Table 2), and thus minimal variation in their respective amino acid sequences, SjTSP-1, -13 and -14 were omitted from further analysis. Considering the variability of SjTSP 3D protein structures across the seven provinces, the structural similarity PCA revealed limited clustering, indicating that across China, SjTSP-2, -8 and -23 variants generally exhibit limited structural conservation. SjTSP-23 sequences from Hunan, Jiangxi and Yunnan as well as SjTSP-8 sequences from Anhui and Zhejiang did, however, demonstrate some structural similarities (Fig. 5). SjTSP-2 provincial sequences revealed a distinct lack of clustering, outlining the extent of the structural variation across parasite populations from the seven provinces.

### Impact of selection on SjTSP structure and function

Predicting the functional effects of amino acid substitutions on the protein-coding regions of the three-variable SjTSPs using PROVEAN revealed that SjTSP-2 and -8 had three function-altering mutations, whereas SjTSP-23 had one (Additional File 3: Fig. S2). Sites with altered functionality in SjTSP-2 were spread across the entire protein, at positions 24, 144 and 169, with the latter two sites residing in the LEL domain as well as the largest epitope in SjTSP-2 (Fig. 4). Similarly, all altered sites in SjTSP-8 (143, 145 & 147) resided within the LEL domain, although are outside predicted epitopes (Fig. 4). In contrast, a single function-altering mutation at site 209 within transmembrane domain 4 (TM4) was predicted for SjTSP-23. Further investigation of genetic divergence between SjTSPs, using TreeSAAP, enabled a comparison of amino acid substitutions between each provincial SjTSP relative to those evolving neutrally. Amino acid substitutions in SjTSP-2, -8 and -23 were predicted to alter a total of 22 physiochemical properties, spanning conformational, chemical, energetic and physical characteristics of amino acids (Table 4). *Z* scores determined by TreeSAAP evaluated the significance of the amino acid replacements, establishing the type of selection pressure acting on each SjTSP. All 22 property changes were significant (Table 4). Five *z* scores reached the highest threshold (> 3.09 = *P* > 0.001); therefore, those property changes were considered useful for inferring those property changes will result from adaptive evolution.

**Table 4:** Significant TreeSAAP *z* scores denoting amino acid physicochemical property alterations across *Schistosoma japonicum* tetraspanin proteins; SjTSP-2, SjTSP-8 and SjTSP-23

Tetraspanin (TSP)	Amino acid physicochemical properties	Magnitude category (*z* scores)			
		5	6	7	8
SjTSP-2	Isoelectric point			2.219 *	1.92 *
	Mean RMS fluctuation displacement			−2.18 *	
	Partial specific volume		2.041 *		
	Chromatographic index		−1.911 *		
	Hydropathy	−1.679 *			
	Composition	−1.739 *			
SjTSP-8	Power to be at the N-terminal				2.385 **
	Refractive index			7.047 ***	
	Composition		7.677 ***		
	Equilibrium constant (ionisation of COOH)		2.349 **		
	Mean RMS fluctuation displacement		2.301 *		
	Average number of surrounding residues		2.228 *		
	Hydropathy		2.210 *		
	Compressibility	5.025 ***			
	Bulkiness	3.139 ***			
	Power to be at the middle of α-helix	2.036 *			
SjTSP-23	Polar requirement			3.238 ***	
	Refractive index			2.575 **	
	Coil tendencies			1.978 *	
	Composition		2.640 **		
	Compressibility	1.674 *	2.407 **		
	Total non-bonded energy		1.862 *		

Magnitude categories denoting ‘radical’ or ‘substantial’ property changes (5–8) are shown; *P < 0.05, **P < 0.01, ***P < 0.001

Isoelectric point, root-mean-square (RMS) fluctuation displacement, chromatographic index and partial specific volume physicochemical properties were predicted to most affect the amino acid biochemistry of SjTSP-2 (Table 4). Based on the TreeSAAP *z* for SjTSP-2, three and four properties were determined to be influenced by positive and purifying selection, respectively. Seven of the nine altered physicochemical properties in SjTSP-8 were considered significant (*P* < 0.05) and radically altered; of these, refractive index and composition were highly significant and under the influence of positive selection and were therefore considered evidence of adaptive molecular evolution [62]. For SjTSP-23, six of the seven altered amino acid properties were predicted to be significant and radically altered, although only polar requirement was deemed to be under positive adaptive selection.

These changes in SjTSP physicochemical properties illustrate the phenotypic impact non-synonymous mutations induce in TSPs from *S. japonicum* populations across China. As established using PROVEAN, most function-altering amino acid changes accumulated within the LEL domain of each variable SjTSP; therefore, the significantly altered physicochemical properties are assumed to also occur in the LEL domain.

### Association of SjTSP genetic variability, antigenicity and antibody binding affinity

**Fig. 6:**
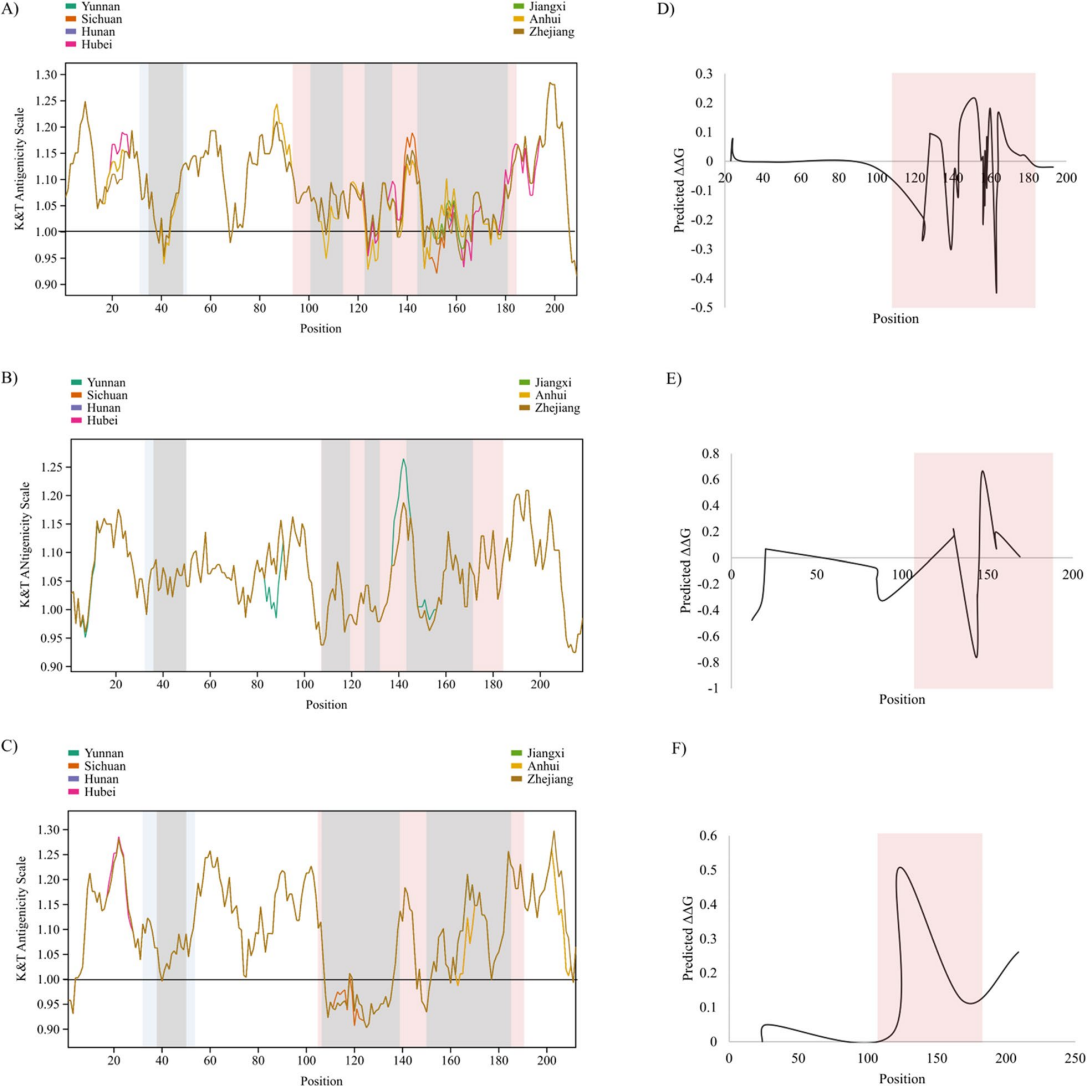
Predicted antigenicity profiles determined for tetraspanins (TSPs) of *Schistosoma japonicum* from Chinese provinces; SjTSP-2 (**A**), SjTSP-8 (**B**) and SjTSP-23 (**C**) and predicted ΔΔG (change in Gibbs free energy) across tetraspanin protein sequences for SjTSP-2 (**D**), SjTSP-8 (**E**) and SjTSP-23 (**F**). SjTSP proteins are considered antigenic where the antigenicity score is > 1 (solid black line). The blue area marks the small extracellular loop domain (SEL) and the red area the large extracellular loop domains (LEL). The grey area denotes BepiPred predicted B-cell epitopes. A negative ΔΔG value corresponded to an amino acid change predicted to reduce affinity, while a positive value was predicted to increase binding affinity. Antigenicity scores were plotted using the Tidyverse package [83] in RStudio [84]

Antigenicity profiles confirmed the substantial antigenic propensity of all seven analysed SjTSPs (Fig. 6A/B/C and Additional File 4: Fig. S3). Interestingly, the exposed LEL domain of these SjTSPs contained distinctly non-antigenic regions (antigenicity scores < 1), particularly in predicted B-cell epitopes. Considerable variation in the antigenicity profiles of provincial SjTSP-2, -8 and -23 was demonstrated. The terminal epitope in SjTSP-2 displayed the most variation in antigenicity between all provincial sequences, whereas the most profound change in antigenicity was demonstrated at residues 141–146 in the LEL domain of SjTSP-8 from Yunnan. In contrast, SjTSP-1, -13, -14 and -25 showed no variation in antigenicity throughout the entire protein (Additional File 4: Fig. S3).

When considering the three SjTSPs displaying variable antigenicity between the provinces, the greatest antigenic fluctuations were predicted within the LEL domain, although certain transmembrane helical regions (e.g. 186-209aa in SjTSP-2) also exhibited variation. Of particular interest is a peak in antigenicity within an area of the LEL domain flanked by predicted B-cell epitopes (140-150aa), which also appears to be a site of substantial antigenic variation between provinces, in SjTSP-2 and -8.

The impact of selection, and resultant amino acid substitutions, is further exemplified through the variability in antibody binding affinity within SjTSP-2, -8 and -23. The predicted antibody binding affinity for each SjTSP is most variable at sites within the LEL domain and is relatively invariable outside the LEL domain, with ΔΔG close to zero (Fig. 6D/E/F). The highest frequency of change occurred across the LEL domain of SjTSP-2, with 10 decreases and 13 increases in affinity (Fig. 6D). A region of SjTSP-2 s LEL domain between residues 141–168 was especially variable, comprising 15 of the 23 predicted antibody binding affinity changes. The largest change in affinity within the LEL domain of SjTSP-2 was a decrease at site 163 (ΔΔG = -0.441), although the greatest change in binding affinity across the three variable SjTSPs was in the LEL domain of SjTSP-8, which experienced a large decrease in affinity at site 143 (ΔΔG = -0.753) and an increase in affinity at site 147 (ΔΔG = 0.665). The greatest alteration in SjTSP-23 binding affinity also occurred in the LEL domain, with a sharp increase at site 123 (ΔΔG = 0.507). Also worth noting is the second largest increase in binding affinity, arising in TM4 at site 209 (ΔΔG = 0.261).

## Discussion

Schistosomes have evolved multiple strategies to evade the host immune system, enabling them to survive in the hostile and complex immunological environment of the host’s vasculature for many years [24]. Surface-exposed proteins, such as tegumental TSPs, are likely fundamental to this strategy [31]. Our findings suggest that schistosome TSPs present at the host-parasite interface are attractive targets for a host immune response and that selection pressures drive polymorphisms in the LEL domain of SjTSPs that impose structural, functional and antibody binding consequences. As such, this diversification acts to reduce the impact of the host’s immune response by limiting antibody recognition, which may reduce their suitability as vaccine targets.

Phylogenetic assessment revealed *S. japonicum* TSPs to be highly divergent, with 27 unique members identified across three major protein subfamilies (CD, CD63 and Uroplakin). The subfamilies resolved here broadly agree with those presented by Wu et al. [32] and Jiang et al. [25], with only minor discrepancies, such as TSP#6 falling into the Uroplakin subfamily rather than the CD63 family [29]. Wu et al. [32] reported that TSPs-1, -16 and -20 fell within the CD9-like group [68], as also found here.

The phylogeographic relationships of SjTSPs-2, -8, -23, and -25 revealed considerable genetic diversity between provincial localities, indicating that between provinces these SjTSPs are evolving independently. This is supported by the ω, MEME and FUBAR analyses, which showed that gene regions encoding the extracellular loops of SjTSP-2, -8 and -23 are particularly influenced by evolutionary selective pressures, likely resulting from exposure of the domains to the host’s immune system. It appears that positive, diversifying selection, acting on specific sites within the LEL domain, promotes variation between TSP proteins from *S. japonicum* isolates from different Chinese provinces. Furthermore, the phylogeographic grouping of provincial SjTSP sequences coupled with similarities in 3D protein structure suggests a link between genetic and structural variation, particularly in SjTSP-8 from Anhui and Zhejiang. Proteins are often functionally constrained, limiting amino acid substitutions and structural variation to maintain function, although clustering of SjTSP-23 from Hunan, Yunnan and Jiangxi, despite a lack of structural similarity from Zhejiang and other provinces, shows that functional constraints and sequence similarity do not eliminate amino acid substitutions from driving structural changes in SjTSPs.

Prolonged selection on the LEL domain has led to formation of a hypervariable region [24, 33], whereby selection appears to favour amino acid changes at certain antibody binding domain codons, as seen between residues 140–147 in the LEL domains of SjTSP-2 and -8. Host-derived selection pressure would promote such hypervariability, thus acting to limit recognition by host antibodies and providing *S. japonicum* with ‘escape’ mutations to facilitate immune evasion [24–26, 33]. This current study revealed distinct variation in SjTSP-LEL antigenicity and antibody binding affinity in SjTSPs-2, -8 and -23 between parasites from different provinces. Antibody binding affinity was predicted to be low in the hypervariable region (140–147) in SjTSP-2-LEL and SjTSP-8-LEL, but was greater thereafter, highlighting the somewhat indiscriminate effect of non-synonymous mutations and resulting amino acid changes on SjTSP immunogenicity. Importantly, the variation identified here may indeed underestimate the total variation in TSP ACGs in *S. japonicum* populations across China, as studies have shown that, at least for TSP2, multiple allelic variants can occur at a single location [24, 30]. Nonetheless, we demonstrate substantial variation between geographical isolates and the evolutionary impact the host could have on the structure and antigenic variability of TSPs from geographically distinct parasite populations.

SjTSP proteins exhibit notable antigenic propensity, with several large peaks in antigenicity predicted across the seven SjTSPs, as previously reported for SmTSP-23 [33]. Findings here support both extracellular loop domains of SjTSPs as highly antigenic, with the LEL containing the highest density of antibody binding sites, confirming the LEL domain of SjTSPs as an attractive target for host immune effector molecules, such as IgG antibodies and B lymphocytes [23, 26, 30]. The LEL domains of variable SjTSPs (TSPs-2, -8 and -23) displayed the greatest fluctuations in antigenicity over the whole protein, supporting the suggestion by Sealey et al. [33] of an association between amino acid variation and antigenic variation in TSP-23, which also extends to SjTSP-2 and -8. Conversely, limited geographical antigenic variability of SjTSP-1, -13 and -14 appears to result from the lack of amino acid variation. Invariable SjTSP-25 antigenicity, despite considerable amino acid variation, however, suggests there is a more complex relationship between amino acid and antigenic variation than previously proposed. It was also predicted that antigenically variable TSPs would contain substantial differences in the length and location of antibody binding sites in the LEL domain [33]. Many sites within the LEL domain of SjTSP-2 and -8 with low antigenicity were associated with a greater occurrence of polymorphic sites. SjTSP-2 displayed evidence of fractured epitopes around the LEL hypervariable region, and SjTSP-23 had shortened epitopes at the terminus of the LEL domain compared to the other SjTSPs. Incidentally, host-derived selection pressures appear to drive a reduction in antigenicity and antibody binding, thereby rendering certain SjTSPs less desirable to host immune components through the accumulation of mutations, highlighting a compelling mechanism of host immunomodulation and evasion by the parasite. Another tactic of host immune evasion and regulation, shown to be facilitated using SjTSP-23, is the inhibition and paralysation of host complement activation through preferential binding to the Fc domain of non-immune IgG [26, 69]. This strategy would require portions of SjTSP-23-LEL to remain attractive targets for IgG, which may be reflected here as a large increase in antibody binding affinity at site 123 in the LEL domain.

Despite evidence of hypervariability and diversifying selection pressures acting on regions encoding SjTSP-2-LEL, high levels of purifying selection were seen across the entire protein, particularly in the transmembrane helical regions. The resulting sequence conservation may be critical for protein localisation and membrane stability and is likely necessary to maintain function and structure. Primarily, selection is episodic, acting more frequently on individual sites than on large gene regions, suggesting that more frequent neutral or purifying selection may obscure phases of adaptive evolution [55]. Minimal local amino acid substitutions can be driven by adaptive evolution if the amino acid changes are biochemically advantageous, as supported by the major functional alterations highlighted by TreeSAAP, and predicted changes in LEL immunogenicity. Use of amino acid substitutions to explore the effect of selection on protein structure and function provided a high degree of resolution and is considered a more sensitive approach than ω [63]. Evolution of antigenic variation, indicated here in certain SjTSPs, is also demonstrated in the pathogen *Bordetella pertussis* (aetiological agent of whooping cough), which has seen a recent resurgence and epidemiological shift to infect older children and adults [70]. The evolution of a novel strain, adapted to express an antigen variant that escapes vaccine-induced immunity, may have caused this shift through sustained selection pressure from host immunity [71]. This evolutionary strategy of immune avoidance is also utilised by the malaria parasite (*Plasmodium* spp.), where antigenic variation, such as seen in PfEMP-1, is the leading mechanism used to evade detection by the host’s immune system [72–74]. The parallels between schistosomes and other pathogenic organisms in their responses to host selection pressures, and mechanisms to drive antigenic variation, could inform us as to how antigenic variation can affect pathogenesis and parasite survivability inside the host in general.

Selection-driven changes in functional properties of TSP-LELs, such as changes in refractive index in SjTSP-8, polar requirement across SjTSP-23 and changes in isoelectric point in SjTSP-2, alter protein folding and might interfere with SjTSP interaction with host molecules, such as antibodies. Antigenic drift, whereby amino acid substitutions lead to changes in antigenicity over time, could also be indicative of balancing selection acting to maintain a greater than expected genetic diversity of SjTSPs through conservation of allelic variants in *S. japonicum* populations. Function-altering mutations within the LEL domain could have direct implications for SjTSP immunogenicity and parasite survival within its definitive host. Although the implications of physicochemical change on antibody binding affinity are seldom investigated, changes in such properties could significantly impact antigen structure, function and immunogenicity. Host immunity differs greatly between species [75], and therefore two different host species could generate contrasting and variable selection pressures [10], leading to different host assemblages promoting the development of host-specific mutations in schistosome TSPs [30, 33, 76]. It could therefore be important to elucidate the variation in ACGs within and between different host species in future studies, not only to increase knowledge regarding the development of an effective anti-schistosome vaccine but also for understanding the varying disease pathology seen in humans infected with schistosomes [77].

Members of the TSP family have consistently demonstrated remarkable genetic diversity in schistosome species [30, 32, 33, 78], which is thought to be responsible, at least in part, for the variable protective efficacy demonstrated so far in TSP vaccine studies [19, 23, 24, 30, 35]. Vaccine development often involves using monomorphic laboratory isolates [79]. However, the geographic variation revealed here for SjTSPs indicates that a diverse range of field-collected parasite isolates should be included in future vaccine trials as they better represent ‘natural’ parasite populations; this strategy would help develop more robust, efficacious vaccines. It has been demonstrated here, for the first time to our knowledge, that SjTSPs with low genetic diversity and invariable antigenicity between provinces, such as SjTSP-1, -13 and -14, could therefore prove more robust vaccine candidates. Several immune-exposed schistosome antigens, however, have demonstrated significant diversifying selection across parasite populations [78]—similar to that indicated here for certain SjTSPs. This leads to a conundrum, as exposed antigens with an apparent propensity for diversification are likely polymorphic in natural, genetically heterogenous parasite populations, and vaccines formulated against them would likely be ineffective. Non-variable antigens, on the other hand, may be internal/non-exposed, rendering them difficult for the immune system to recognise and mount an effective response against [78]. As little is known about the cellular localisation or function of SjTSP-13, -14 and -25, further studies on these TSPs are warranted. Regardless, vaccine development based on a single polymorphic form of a TSP would likely offer low efficacy against natural parasite populations. It has recently been proposed that the endeavour to develop a single-target vaccine has significant limitations, with evidence suggesting a multi-target, multivalent DNA or mRNA vaccine would provide the best protection [80, 81]. Furthermore, any vaccine would require deployment alongside, and integration within, existing control strategies to be suitably effective [17, 81].

## Conclusion

Despite significant reductions in infections across China, *S. japonicum* remains endemic in humans and animal reservoirs in several provincial regions [13], and the risk of re-emergence remains a legitimate concern [9]. It seems that the development of an effective anti-schistosome vaccine, intended to reach end-game elimination targets in China [8], has been hampered by the knowledge gap associated with the genetic diversity and antigenic variability of vaccine candidate antigens within and between parasite populations. To begin to address this, measuring the genetic diversity and evolutionary processes that effect vaccine targets, such as SjTSPs, could be crucial in identifying robust vaccine targets as well as potential mechanisms employed by the parasite to evade the host immune response. As such, these findings are not only useful in evaluating the use of SjTSPs as vaccine candidates, but they also provide an analytical framework with which to explore genetic and antigenic variability in other schistosome vaccine candidates. Findings reported here show that SjTSPs' genetic and antigenic variability between geographically distinct parasite isolates could feasibly influence the effectiveness of the host immune response, impacting vaccine efficacy and representing a challenge for vaccine development against *S. japonicum*. It is therefore recommended that robust evaluation of vaccine target genetic and antigenic variability in natural parasite populations should be a prerequisite for any vaccine development campaign in addition to further immunological work experimentally validating alterations in TSP antigenicity and antibody binding affinity detected here.

## Supplementary Information

**Additional file 1: Fig S1.** Map of China outlining the seven parasite populations (red) used in this study, which were sampled by Young et al. [34], and provincial boundaries (white). In relation to sampling locations reported by Young et al. [34]; Ya’an (Sichuan) represents Tianquan, Jiujiang (Jiangxi) represents Yongxia, Chizhou (Anhui) represents Guichi, and Jiaxing (Zhejiang) represents Jiashan. Sampling location names were changed to reflect the proper district-level classifications. Created using QGIS v3.26.3 [87].

**Additional file 2: Table S1.** Identification of individual nucleotide bases under selection in SjTSP-2, SjTSP-8 and SjTSP-23 using MEME and FUBAR.

**Additional file 3: Fig S2.** Investigating site-specific functional alterations resulting from amino acid changes using PROVEAN. SjTSP-2 (A), SjTSP-8 (B), SjTSP-23 (C), SjTSP1 (D), SjTSP-13 (E), SjTSP-14 (F), SjTSP-25 (G). A PROVEAN score < –2.5, denoted by the back horizontal line, outlines an amino acid change that will induce a functional alteration at that site.

**Additional file 4: Fig. S3.** Identical predicted antigenicity profiles determined for tetraspanins (TSPs) of *Schistosoma japonicum* from Chinese provinces; SjTSP-1 (A), SjTSP-13 (B), SjTSP-14 (C), SjTSP-25 (D). SjTSP proteins are considered antigenic where the antigenicity score is > 1 (solid black line).

## Data Availability

In silico study based on published *Schistosoma japonicum* genome data as described in the methods.
