# Supplementary figures and images for: Evolution of tetraspanin antigens in the zoonotic Asian blood fluke Schistosoma japonicum
Source: Parasit Vectors. 2023 Mar 14;16:97. doi: 10.1186/s13071-023-05706-3 (PMC10012309; doi:10.1186/s13071-023-05706-3)

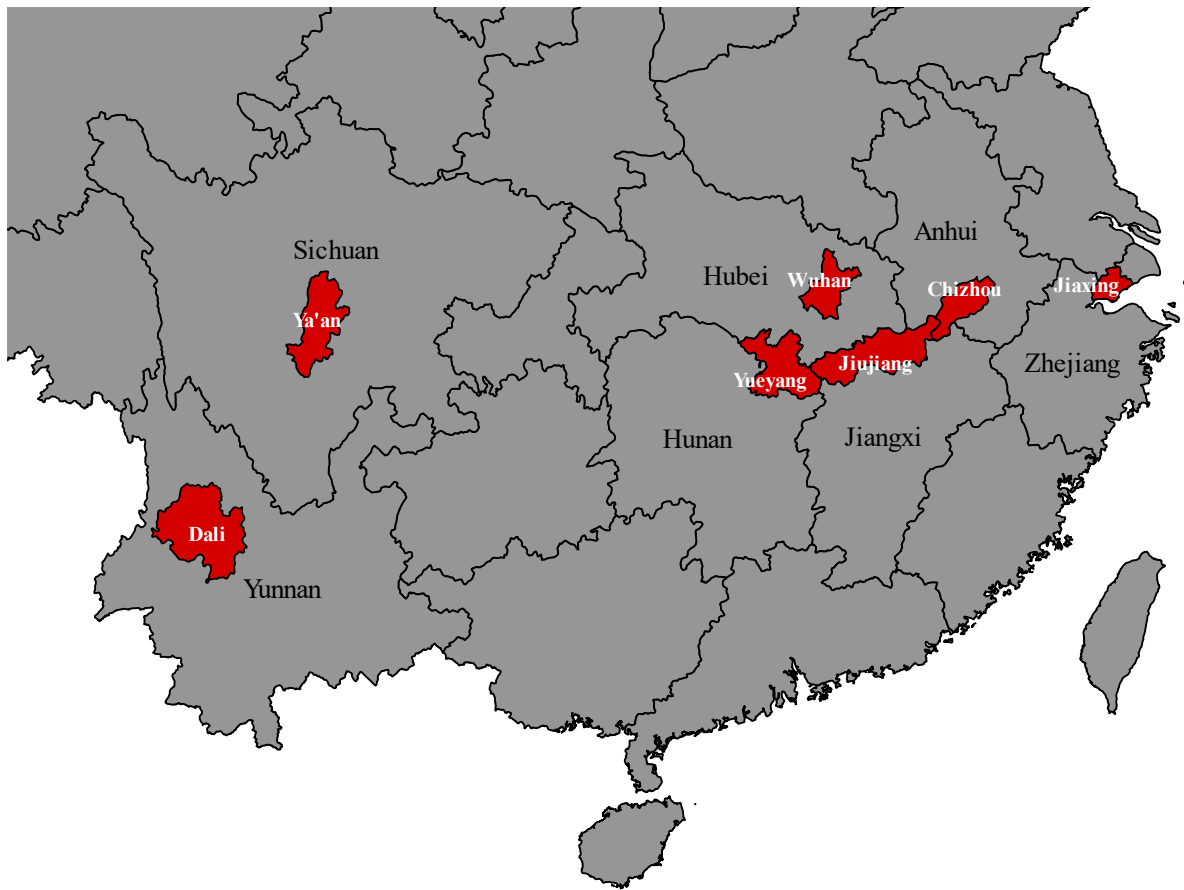

Supplement: Supplementary file 1 — Additional file 1: Fig S1. Map of China outlining the seven parasite populations (red) used in this study, which were sampled by Young et al. [34], and provincial boundaries (white). In relation to sampling locations reported by Young et al. [34]; Ya’an (Sichuan) represents Tianquan, Jiujiang (Jiangxi) represents Yongxia, Chizhou (Anhui) represents Guichi, and Jiaxing (Zhejiang) represents Jiashan. Sampling location names were changed to reflect the proper district-level classifications. Created using QGIS v3.26.3 [87]. [file 13071_2023_5706_MOESM1_ESM.pdf]

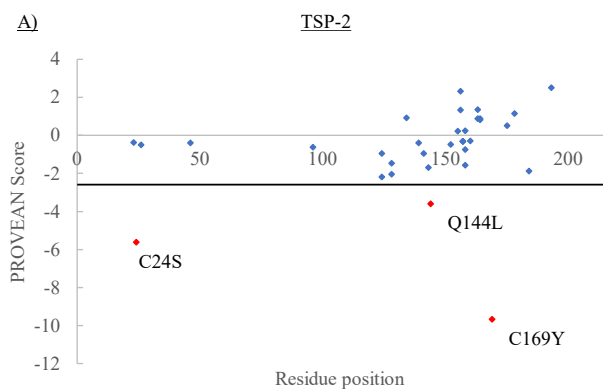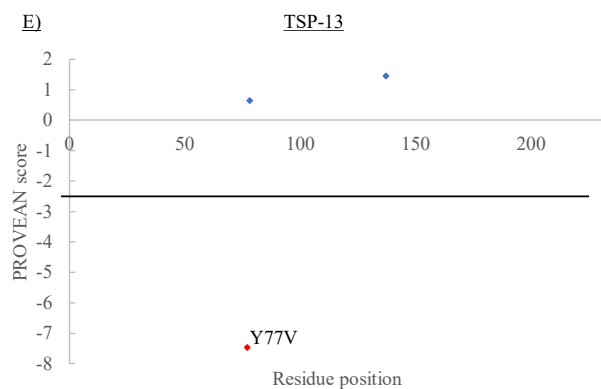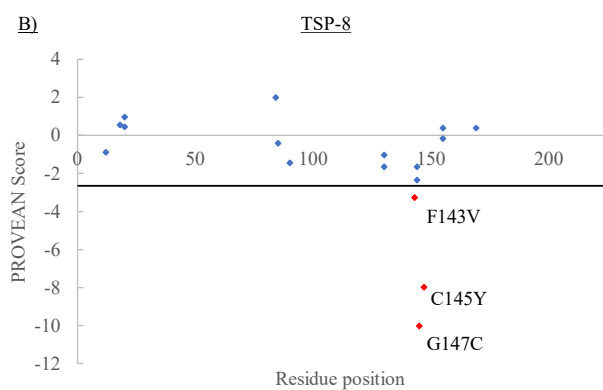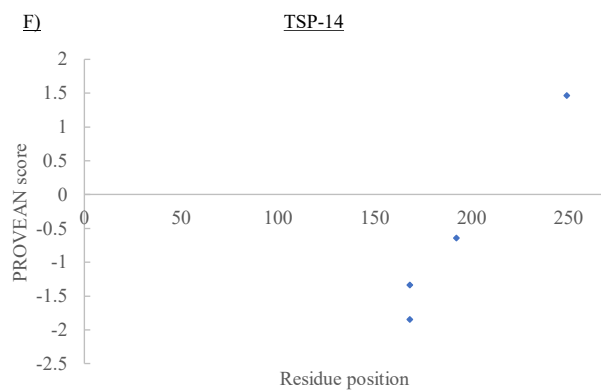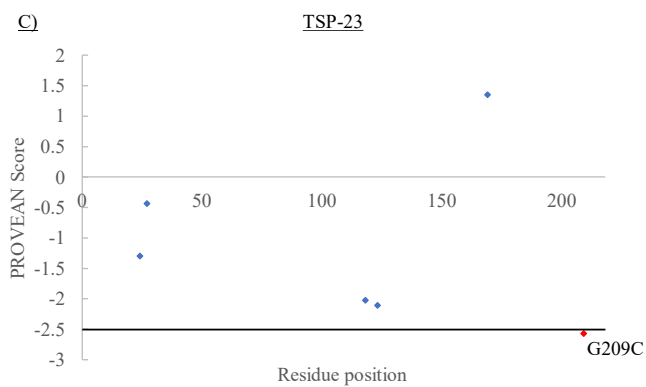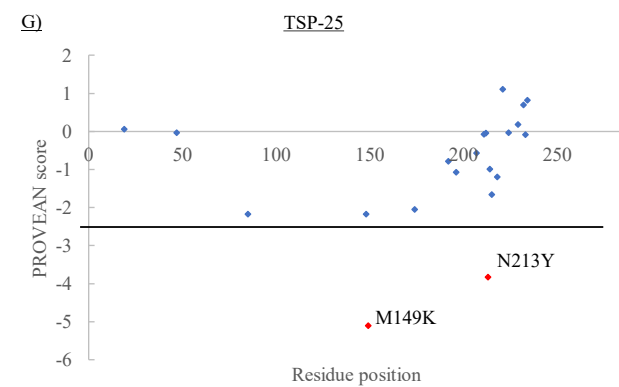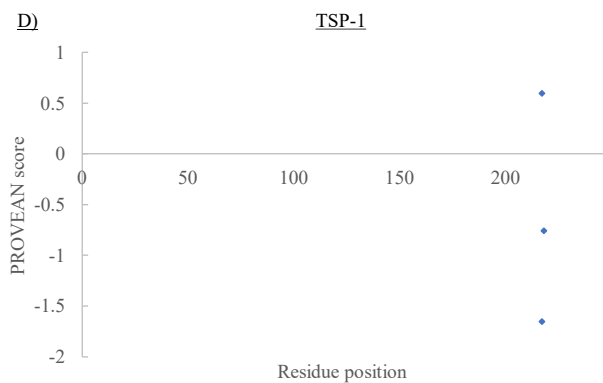

Supplement: Supplementary file 3 — Additional file 3: Fig S2. Investigating site-specific functional alterations resulting from amino acid changes using PROVEAN. SjTSP-2 (A), SjTSP-8 (B), SjTSP-23 (C), SjTSP1 (D), SjTSP-13 (E), SjTSP-14 (F), SjTSP-25 (G). A PROVEAN score < –2.5, denoted by the back horizontal line, outlines an amino acid change that will induce a functional alteration at that site. [file 13071_2023_5706_MOESM3_ESM.pdf]
