# Supplementary material for: Evolution of tetraspanin antigens in the zoonotic Asian blood fluke Schistosoma japonicum
Source: Parasit Vectors. 2023 Mar 14;16:97. doi: 10.1186/s13071-023-05706-3 (PMC10012309; doi:10.1186/s13071-023-05706-3)
Supplement: Supplementary file 4 — Additional file 4: Fig. S3. Identical predicted antigenicity profiles determined for tetraspanins (TSPs) of Schistosoma japonicum from Chinese provinces; SjTSP-1 (A), SjTSP-13 (B), SjTSP-14 (C), SjTSP-25 (D). SjTSP proteins are considered antigenic where the antigenicity score is > 1 (solid black line). [file 13071_2023_5706_MOESM4_ESM.pdf]

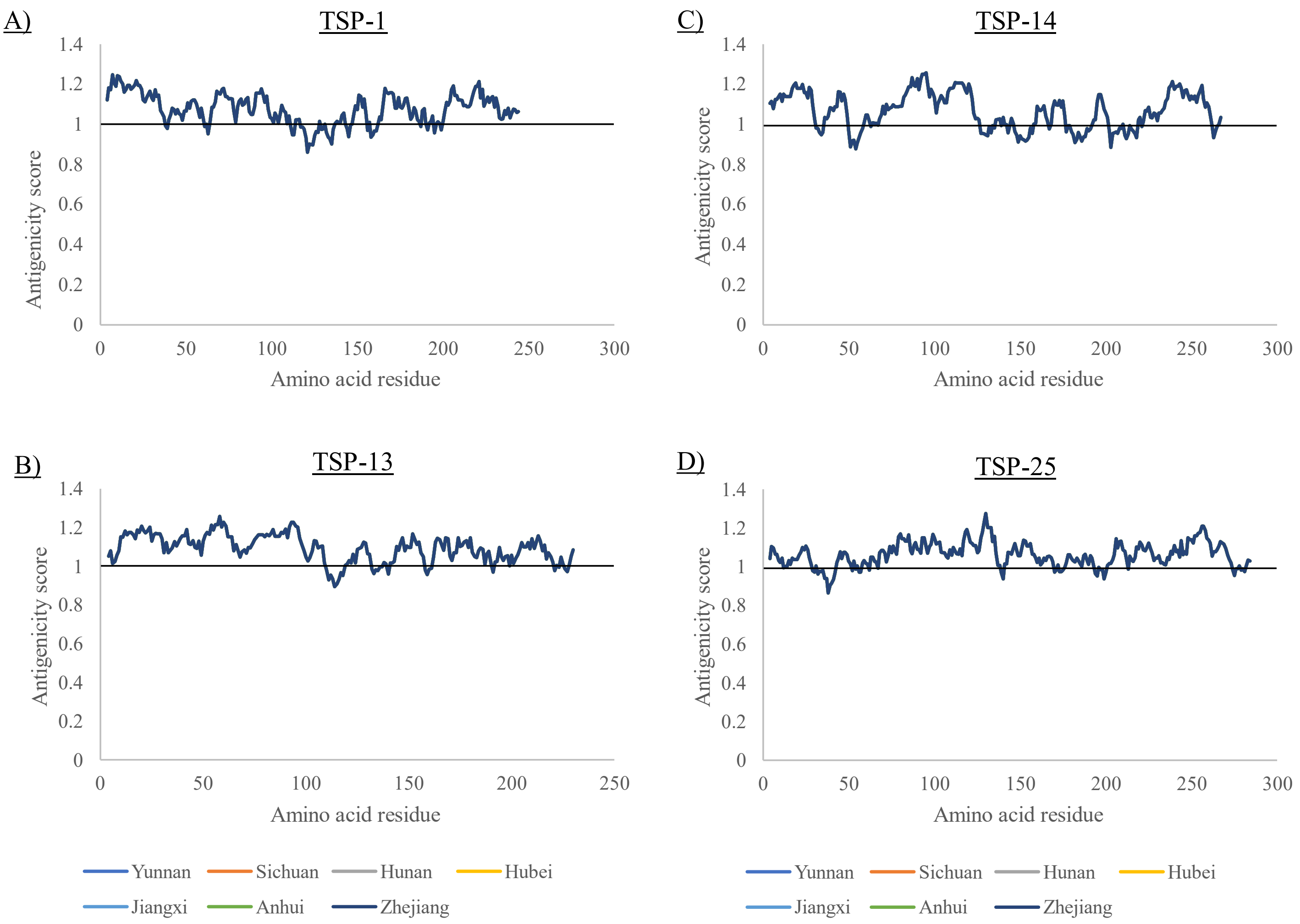

A)
TSP-1
Antigenicity score
Amino acid residue
B)
TSP-13
Antigenicity score
Amino acid residue
C)
TSP-14
Antigenicity score
Amino acid residue
D)
TSP-25
Antigenicity score
Amino acid residue
Yunnan
Sichuan
Hunan
Hubei
Jiangxi
Anhui
Zhejiang
Yunnan
Sichuan
Hunan
Hubei
Jiangxi
Anhui
Zhejiang
